# Trends in deaths and disability-adjusted life-years of stroke attributable to low physical activity worldwide, 1990–2019

**DOI:** 10.1186/s12889-023-17162-w

**Published:** 2023-11-14

**Authors:** Jun-xiao Li, Qiong-qiong Zhong, Shi-xiang Yuan, Feng Zhu

**Affiliations:** 1 Central Laboratory, Guangzhou Twelfth People’s Hospital, Guangzhou, China; 2 Departments of Public Health and Preventive Medicine, Jinan University, Guangzhou, China; 3 Department of Neurosurgery, Guangzhou Twelfth People’s Hospital, Guangzhou, China

**Keywords:** Physical activity, Stroke, Disease burden, Disability-adjusted life years, Mortality

## Abstract

**Background:**

*Low physical activity (LPA) is linked to the risk of stroke, but the disease burden of stroke attributable to LPA needs to be understood to develop effective preventive strategies.* We aim to assess spatiotemporal trends in the global burden of stroke attributable to LPA from 1990 to 2019.

**Methods:**

Based on the Global Burden of Disease, Injuries, and Risk Factors Study, our research examined deaths, the Disability-Adjusted Life Years (DALYs), the Age-Standardized Mortality Rate (ASMR), the Age-Standardized DALY Rate (ASDR), and the Estimated Annual Percentage Change (EAPC) for stroke attributable to LPA.

**Results:**

Deaths and DALYs were on the rise worldwide from 1990 to 2019, with increases of 72.72% for the former and 67.41% for the latter; ASMR and ASDR decreased, with the ASMR-related EAPC of -1.61 (95% CI:-1.71–-1.5) and ASDR-related EAPC of -1.35 (95% CI:-1.43–-1.27); females had more numbers of deaths and DALYs, and the majorities of deaths and DALYs were shared by those aged ≥ 70. The highest burden rates were shared by North Africa, the Middle East, and Tropical Latin America; the ASMR-related EAPC was associated with the ASMR in 1990 (R = -0.26, *P* < 0.001) and the Socio-Demographic Index (SDI) across different countries in 2019 (R = -0.61, *P* < 0.001), respectively, and such patterns were similar to what ASDR and the ASDR-related EAPC had; the Human Development Index (HDI) in 2019 was associated with the ASMR-related EAPC (R = 0.63, *P* < 0.001) and the ASDR-related EAPC across different countries (R = -0.62, *P* < 0.001), respectively.

**Conclusions:**

Globally, *deaths and DALYs of stroke attributable to LPA were on the rise, although their age-standardized rates presented downward over the past three decades*; *the burden of stroke attributable to LPA showed upward trends especially in those aged* ≥ *70 and females in the regions of East Asia, North Africa, and the Middle East, which need more attention to the effects of physical activity on health interventions*.

## Introduction

Stroke, one of the leading causes of mortality and morbidity, has become a public health problem in the world, according to the Global Burden of Diseases, Injuries, and Risk Factors Study (GBD) in 2019 [1, 2]. Among a series of concerning data on incidence, deaths, and age-standardized rates (ASR) of the Disability-Adjusted Life-Years (DALYs), the age-standardized rates of stroke decreased from 1990 to 2019, with decreases of 17.0% for incidence, 36.0% for mortality, 6.0% for prevalence, and 36.0% for DALYs; stroke remained the second-leading cause of deaths and the third-leading cause of the death and disability combined worldwide in 2019 [3]. The estimated direct and indirect costs of stroke in 2017 worldwide were approximately 1.12% of the global gross domestic product (GDP) [4]. *Physical activity is linked to stroke* [5–7] and it is strongly associated with breast cancer, diabetes, cardiovascular disease, and other conditions [8–11]. The US Activity Guidelines recommended the maintenance of appropriate physical activities and *the limitation* of sedentary activities [12], which should benefit almost all teens, adults, seniors, and pregnant women. However, physical inactivity (or Low Physical Activity, LPA) *worldwide has been occurring since 2001* [13], and a policy on strengthening physical activity should be formulated and implemented *to control* the risk of unhealthy behavioral risk factors.

Recent evidence reveals that unhealthy behavioral risk factors, such as a sedentary lifestyle or LPA, tend to increase with development [14]. Thus, it is obvious that the corresponding burden *will concentrate in* some developing countries with large populations, such as Brazil, Russia, India, and China (BRIC), where *the populations aged* > *60 years are predicted t*o at least double by 2050 [15, 16]; LPA showed an unequivocal association with the risk of stroke, with the ranks of 18/19 for all strokes and 14/19 for ischaemic stroke in the list of risk factors based on a total number of all ages DALYs worldwide in 2019 [3], the disease burden of stroke attributable to LPA should be in urgent need for a systematic assessment. *Nevertheless,* the current estimates of the global burden of stroke attributable to LPA and its temporal trends are still incompletely understood. Accurate and up-to-date estimates of this burden a*re important in* planning research and the resulting evidence-based strategies for stroke prevention and management.

GBD provides an opportunity to incorporate newly available datasets, enhance method performance and standardization, and be in response to changes in scientific knowledge. Here, we aimed *to estimate spatiotemporal trends in the deaths and DALY of stroke attributable to LPA globally, by regions, HDIs, SDIs, countries, gender, and age groups* from 1990 to 2019 for guiding allocations of sports resources, bettering the strategies *in the promotion on p*hysical activity, and lowering the disease burden of stroke.

## Methods

### Data source and study sample

Study data were taken from the Global Health Data Exchange (GHDx, http://ghdx.healthdata.org/gbd-results-tool) of the Institute for Health Metrics and Evaluation (IHME) at the University of Washington, the World Health Organization (WHO), and members of the Global GBD Collaborative Group. GBD recorded 369 diseases and 87 risk factors in 1990–2019 and listed 204 countries and territories and 21 regions, with newcomers of the Cook Islands, Monaco, San Marino, Nauru, Niue, Palau, Saint Kitts and Nevis, Tokelau, and Tuvalu; thus, all the World Health Organization (WHO) members were given in GBD 2019 [2]. All methods of this GBD study were carried out in accordance with the Guidelines for Accurate and Transparent Health Estimates Reporting (GATHER) guidelines.

### Low physical activity and disease identification

Physical activity in GBD was measured for those aged ≥ 25, and their life conditions (leisure/recreation, work/home, and transportation) were included in terms of the frequency, duration, and intensity of each physical activity lasting at least ten minutes. The Metabolic Equivalent (MET), a ratio of the working metabolic rate to the resting metabolic rate, was introduced. Based on total MET-minutes per week, physical activity was categorized into inactivity (< 600 MET-minutes per week), low activity (600–3,999 MET-minutes per week), moderate activity (4,000–7,999 MET-minutes per week), and high activity (≥ 8,000 MET-minutes per week). Theoretically, the minimum exposure to physical inactivity was 3,000–4,500 MET-minutes per week [17]; thus, LPA was defined as < 3,000 MET-minutes per week.

*Details of the GBD 2019 eligibility criteria, the literature search strategy, and data extraction are described in detail elsewhere. In brief, stroke* was defined by the WHO criteria and was estimated based on the International Statistical Classification of Diseases and Related Health Problems (ICD), GBD 2019, and the Cause List Mapped to the ICD codes of the I60–I62, I62.9–I64, I64.1, I65–I69.998, Z82.3, and G45–46.8 [18] as follow: rapidly developing clinical signs of focal (at times global) disturbance of cerebral function lasting more than 24 h or leading to death and of presumed vascular origin; additionally, ischemic attack, subarachnoid hemorrhage, and subarachnoid hemorrhage were included. *Stroke attributable to LPA was based on defining stroke by combining the distribution of exposure to risk-related LPA* [19].

### Disease burden measurement

We collected *the aggregated countries’ data* and used analytic tools on the GBD website. Disease burdens were assessed with a range of indicators, including deaths, DALY, and the Age-Standardized Rates (ASR; the Age-Standardized Mortality Rate (ASMR) and the Age-Standardized DALY Rate (ASDR)). ASR was calculated with a no-weighted mean of GBD year’s age-specific proportional distributions for national locations with populations greater than 5 million in GBD year to update the world population age standard. In brief, ASR was generated from several parameters, including a summing up age-standardized rate (a_*i*_, wherein *i* is the *i*^th^ age class), a number (or the weight) of persons (*w*_*i*_) in the same age subgroup *i* (a reference of the standard population), and a dividend of *summing up the standard population weight* [20]:

$$\mathrm{ASR }=\frac{{\sum }_{i=1}^{A}{a}_{i}{w}_{i}}{{\sum }_{i=1}^{A}{a}_{i}}\times \mathrm{100,000}.$$

According to this formula, we presented ASMR per 100,000 person-years, and ASDR per 100,000 people with the direct method of standardization and the WHO’s standard population. The Estimated Annual Percentage Change (EAPC), which was widely used to describe a trend of ASR, was calculated as follows:

$$\mathrm{y}=\mathrm{\alpha }+{\upbeta }_{\mathrm{x}}+\upvarepsilon ;\mathrm{EAPC}=100 \times [\mathrm{exp }(\upbeta )-1].$$

*Where y is equal to the natural logarithm of ASR* and *x* corresponds to the calendar year.

EAPC and its 95% confidence interval (CI) were estimated using a linear regression model. ASRs would be upward, downward, and stable if EAPCs with the 95% CI were > 0, < 0, and = 0. The sociodemographic index (SDI) from the GHDx (http://ghdx.healthdata.org) is a composite index of per capita income, educational attainment, and total fertility rate of all areas, identifying the socio-demographic development status of countries or other geographic regions. It is calculated as the geometric mean on a scale of 0 to 1, by which all countries are divided into five groups: *low (0–0.455), low-middle (0.455–0.608), middle (0.608–0.690), middle-high (0.690–0.805), and high (0.805–1) * [21]. Data from 204 countries and territories, 5 SDIs, and 21 GBD regions, in terms of epidemiological and geographical conditions, were all available and used to estimate [2]. The Human Development Index (HDI) is a summary measurement of an average achievement in the key dimensions of human development, including a long and healthy life, being knowledgeable, and having a decent standard of living *and can be downloaded* from the United Nations Development Program (http://hdr.undp.org/en/data).

### Statistical analysis

Deaths, ASMR, DALY, and ASDR *were quantified as the disease burdens at* global, regional, and national levels. The regions and the territories were also divided into 21 groups by region and 15 groups by age. All values for each metric were represented as the Uncertainty Intervals (UIs) using the 25^th^ and the 975^th^ ordered 1,000 draw values of the posterior distribution. Relationships between SDIs and ASRs from 1990 to 2019 were evaluated. With the social development and health outcomes by SDI in 2019 and the baseline level of disease burden by the ASR in 1990, the HDI in 2019 could reflect a national condition in all health sources. *To explore the influential factors for EAPC, the Pearson correlation analysis was used to assess the association between EAPC and SDI in 2019, ASR in 1990, and HDI in 2019, respectively* [22]. All analyses in this study were performed using the R software (R core team, the version of 4.1.3, Vienna, Austria), and statistical significance was defined as *P* < 0.05.

## Results

### The global burden of stroke attributable to LPA

**Fig. 1:**
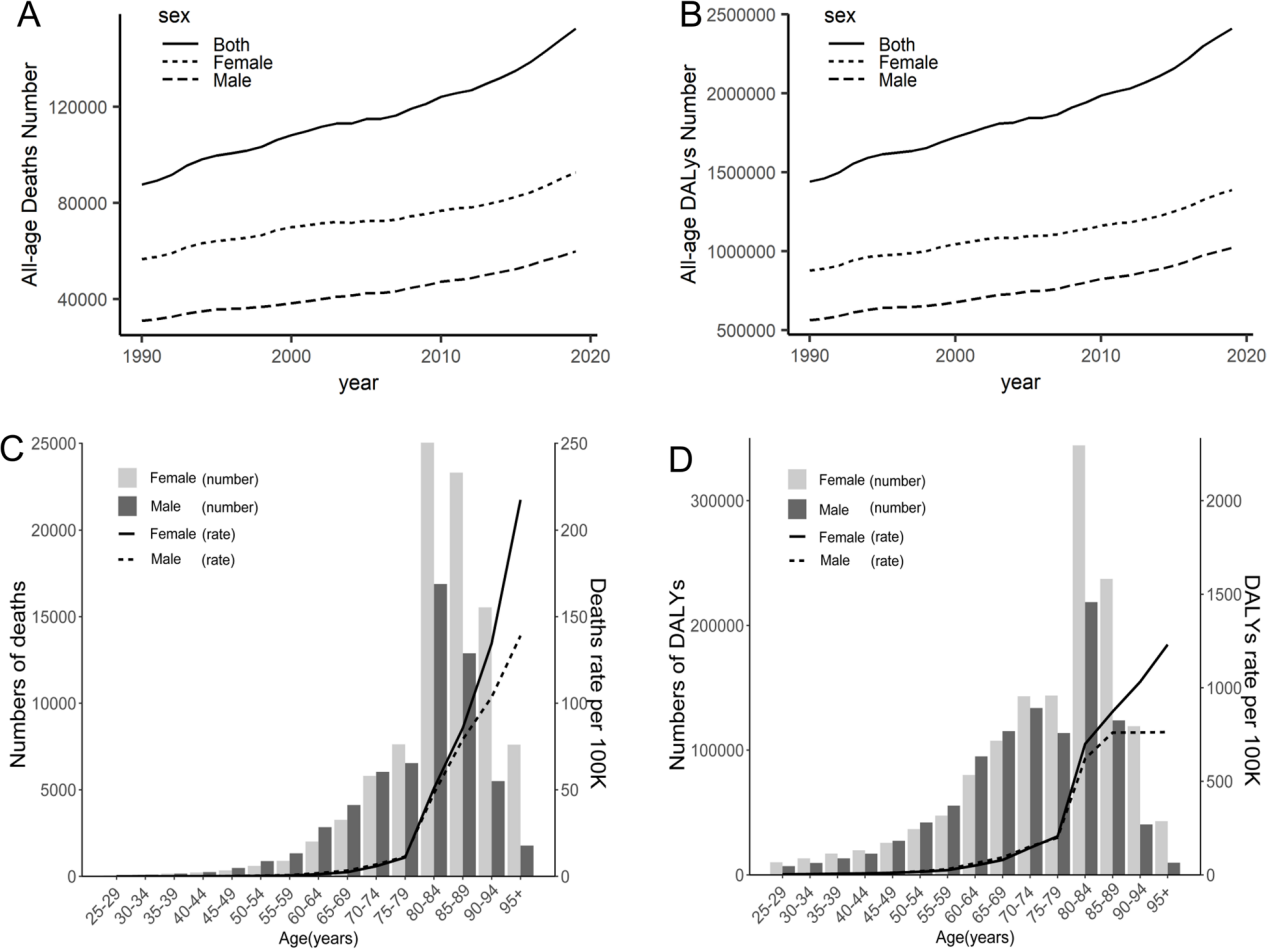
Global deaths (**A**) and DALY (**B**) of stroke attributable to LPA in all ages, 1990–2019; global numbers and rates of deaths (**C**), DALY (**D**) of stroke attributable to LPA by age and gender, 2019. DALY: disability-adjusted life-years; LPA: low physical activity

Globally, deaths of stroke attributable to LPA were on the rise in 1990–2019, with an increase of 72.72%, wherein males showed a higher growth (93.55%) than females (63.16%), but females had more death numbers (0.09 million, 95% UI: 0.02–0.23 million) than males (0.06 million, 95% UI: 0.01–0.17 million) in 2019 (Fig. 1A and Table 1); similarly, the DALYs of stroke attributable to LPA were on the rise in 1990–2019, with an increase of 67.41%, wherein males showed a higher growth (81.17%) than females (58.68%), but females had a higher DALYs (1.39 million, 95% UI: 0.28–3.49 million) than males (1.02 million, 95% UI: 0.15–2.97 in 2019 (Fig. 1B and Table 1).

**Table 1:** Deaths, the DALYs and their corresponding ASRs in 1990 and 2019 and the EAPC in the ASRs from1990 to 2019

	Deaths				The DALYs				The EAPC (1990–2019)	
	no. × 10^5^ (95% UI) in 1990	age-standardized no. × 10^−5^ (95% UI) in1990	no. × 10^5^ (95% UI) in 2019	age-standardized no. × 10 − 5 (95% UI) in 2019	no. × 10^5^ (95% UI) in 1990	age-standardized no. × 10 − 5 (95% UI) in 1990	no. × 10^5^ (95% UI) in 2019	age-standardized no. × 10 − 5 (95% UI) in 2019	the ASMR no. (95% CI)	the ASDR no. (95% CI)
**Global**	0.88 (0.16–2.31)	3.09 (0.55–8.09)	1.52 (0.3–3.92)	2.08 (0.41–5.33)	14.39 (2.4–39.31)	43.55 (7.56–117.17)	24.09(4.33–63.78)	31.16 (5.69–82.02)	-1.61(-1.71–1.5)	-1.35(-1.43–1.27)
**Sex**										
Female	0.57 (0.11–1.4)	3.24 (0.64–7.94)	0.93 (0.19–2.27)	2.1 (0.44–5.15)	8.76 (1.65–22.6)	45.5 (8.73–115.81)	13.9 (2.83–34.9)	31.6 (6.45–79.7)	-1.75(-1.87–1.63)	-1.46(-1.55–1.36)
Male	0.31 (0.04–0.91)	2.74 (0.37–8.06)	0.6 (0.09–1.68)	1.99 (0.31–5.52)	5.63 (0.73–16.9)	39.6 (5.42–113.4)	10.2 (1.52–29.7)	29.9 (4.52–85.1)	-1.3(-1.37–1.23)	-1.13(-1.19–1.07)
**SDI**										
High SDI	0.26 (0.04–0.73)	2.55 (0.36–7.12)	0.26 (0.04–0.68)	1.06 (0.17–2.83)	3.69 (0.51–10.71)	35.12 (4.87–101.83)	3.6 (0.54–9.92)	17.44 (2.53–48.12)	-3.49(-3.66–3.31)	-2.8(-2.97–2.63)
High-middle SDI	0.34 (0.07–0.89)	4.41 (0.89–11.25)	0.53 (0.11–1.35)	2.75 (0.56–6.97)	5.45 (0.97–14.82)	60.25 (11.38–159.34)	7.65 (1.44–20.07)	38.45 (7.27–100.71)	-1.92(-2.11–1.73)	-1.84(-2–1.68)
Middle SDI	0.16 (0.03–0.44)	2.66 (0.49–6.99)	0.46 (0.08–1.22)	2.48 (0.47–6.52)	3.18 (0.52–8.89)	40 (6.9–107.25)	7.98 (1.33–21.98)	37.6 (6.64–102.39)	-0.36(-0.49–0.23)	-0.33(-0.42–0.25)
Low-middle SDI	0.08 (0.02–0.21)	2.57 (0.55–6.2)	0.23 (0.05–0.55)	2.34 (0.52–5.64)	1.6 (0.3–4.25)	36.47 (7.32–90.6)	3.8 (0.78–9.81)	33.4 (7.26–83.02)	-0.36(-0.5–0.22)	-0.31(-0.42–0.21)
Low SDI	0.02 (0–0.06)	1.77 (0.35–4.45)	0.05 (0.01–0.14)	1.69 (0.37–4.21)	0.46 (0.08–1.36)	26.46 (4.9–70.41)	1.04 (0.19–2.91)	25.17 (4.97–66.78)	-0.02(-0.14–0.1)	-0.03(-0.12–0.07)
**Region**										
Andean Latin America	0 (0–0)	0.96 (0.1–3)	0 (0–0.01)	0.73 (0.08–2.25)	0.03 (0–0.08)	13.92 (1.54–45)	0.06 (0.01–0.17)	10.37 (1.15–31.69)	-0.91(-1.07–0.76)	-1.08(-1.26–0.89)
Australasia	0.01 (0–0.01)	2.75 (0.36–7.23)	0.01 (0–0.02)	1.3 (0.19–3.2)	0.07 (0.01–0.21)	34.15 (4.49–95.82)	0.09 (0.01–0.23)	16.39 (2.31–43.06)	-3.14(-3.35–2.94)	-3.01(-3.17–2.86)
Caribbean	0.01 (0–0.02)	3.26 (0.5–7.81)	0.01 (0–0.03)	2.88 (0.43–6.65)	0.11 (0.02–0.28)	45.47 (7.02–115.61)	0.21 (0.03–0.52)	41.4 (6.49–101.95)	-0.35(-0.44–0.26)	-0.23(-0.33–0.13)
Central Asia	0.01 (0–0.03)	2.82 (0.56–7.55)	0.01 (0–0.04)	3.27 (0.66–8.64)	0.17 (0.03–0.52)	41.73 (8.14–121.31)	0.26 (0.05–0.77)	45.51 (8.54–127.74)	0.11(-0.18–0.4)	-0.12(-0.42–0.18)
Central Europe	0.05 (0.01–0.13)	4.27 (0.91–11.14)	0.07 (0.01–0.17)	2.95 (0.64–7.59)	0.8 (0.15–2.24)	60.16 (11.85–166.43)	0.9 (0.19–2.37)	39.78 (8.1–104.77)	-1.63(-1.8–1.46)	-1.76(-1.91–1.61)
Central Latin America	0.01 (0–0.02)	1.05 (0.13–3.36)	0.01 (0–0.04)	0.66 (0.08–2.07)	0.12 (0.01–0.41)	16.03 (1.87–51.76)	0.22 (0.03–0.69)	9.86 (1.17–30.91)	-2.08(-2.31–1.85)	-2(-2.22–1.77)
Central Sub-Saharan Africa	0 (0–0.01)	1.95 (0.33–5.41)	0.01 (0–0.02)	2.11 (0.39–5.67)	0.05 (0.01–0.15)	31.34 (4.87–87.39)	0.12 (0.02–0.36)	32.03 (5.49–88.24)	0.23(0.18–0.27)	0.02(-0.02–0.06)
East Asia	0.14 (0.02–0.39)	2.84 (0.46–7.69)	0.39 (0.06–1.11)	2.52 (0.41–6.94)	2.75 (0.42–8.14)	42.3 (6.71–113.68)	6.75 (1.05–19.67)	37.81 (5.97–106.95)	-0.71(-0.93–0.49)	-0.69(-0.83–0.56)
Eastern Europe	0.12 (0.02–0.33)	5.51 (1.14–14.65)	0.14 (0.03–0.38)	4.09 (0.89–10.62)	1.83 (0.36–5.38)	73.48 (14.43–212.75)	1.88 (0.39–5.18)	52.47 (10.86–145.86)	-1.53(-2.01–1.04)	-1.63(-2.07–1.19)
Eastern Sub-Saharan Africa	0 (0–0.01)	0.68 (0.13–2.39)	0.01 (0–0.03)	0.78 (0.15–2.62)	0.07 (0.01–0.29)	11.49 (2.23–44.04)	0.16 (0.03–0.66)	12.46 (2.47–46.29)	0.58(0.53–0.62)	0.34(0.32–0.37)
High-income Asia Pacific	0.05 (0.01–0.14)	3.05 (0.35–9.21)	0.06 (0.01–0.18)	0.92 (0.11–2.66)	0.72 (0.08–2.29)	41.04 (4.5–128.67)	0.84 (0.09–2.51)	15.08 (1.63–45.91)	-4.56(-4.77–4.35)	-3.82(-4–3.65)
High-income North America	0.05 (0.01–0.16)	1.47 (0.18–4.3)	0.05 (0.01–0.16)	0.67 (0.08–2.08)	0.85 (0.1–2.55)	23.21 (2.74–69.47)	0.81 (0.09–2.54)	12.43 (1.36–38.85)	-3.28(-3.64–2.92)	-2.54(-2.77–2.3)
North Africa and Middle East	0.06 (0.01–0.14)	5.79 (1.32–12.19)	0.18 (0.04–0.37)	5.6 (1.34–11.58)	1.26 (0.25–3.02)	90.62 (19.1–204.65)	3.5 (0.69–8.14)	91.71 (18.75–203.41)	0.05(-0.04–0.15)	0.19(0.1–0.27)
Oceania	0 (0–0)	1.75 (0.28–4.63)	0 (0–0)	1.83 (0.33–4.76)	0.01 (0–0.02)	30.96 (4.69–83.98)	0.02 (0–0.05)	32.35 (5.01–86.03)	0.03(-0.03–0.08)	0.05(-0.01–0.11)
South Asia	0.06 (0.01–0.16)	2.2 (0.44–5.7)	0.16 (0.03–0.41)	1.75 (0.38–4.38)	1.13 (0.19–3.21)	29.81 (5.62–78.29)	2.6 (0.51–7.12)	23.27 (4.73–60.92)	-1.01(-1.32–0.7)	-0.91(-1.15–0.68)
Southeast Asia	0.03 (0–0.1)	2.15 (0.28–6.46)	0.1 (0.01–0.32)	2.41 (0.32–7.31)	0.67 (0.08–2.17)	33.32 (4.23–102.76)	1.89 (0.25–5.93)	37.15 (4.99–113.26)	0.56(0.43–0.69)	0.49(0.4–0.58)
Southern Latin America	0 (0–0.01)	0.93 (0.14–3.2)	0.01 (0–0.02)	0.62 (0.09–1.95)	0.06 (0.01–0.23)	13.99 (2.19–53.06)	0.08 (0.01–0.24)	8.94 (1.27–29.11)	-1.21(-1.41–1.01)	-1.37(-1.55–1.19)
Southern Sub-Saharan Africa	0 (0–0.01)	2.19 (0.41–5.59)	0.01 (0–0.03)	2.76 (0.55–6.93)	0.08 (0.01–0.23)	34.97 (5.95–92.33)	0.18 (0.03–0.49)	39.91 (7.25–102.72)	1.3(0.68–1.92)	0.96(0.36–1.57)
Tropical Latin America	0.05 (0.01–0.1)	7.43 (1.3–15.42)	0.08 (0.02–0.16)	3.84 (0.92–7.35)	0.82 (0.12–1.98)	108.75(17.24–243.98)	1.24 (0.26–2.54)	54.8 (11.78–110.74)	-2.18(-2.33–2.04)	-2.31(-2.45–2.18)
Western Europe	0.2 (0.03–0.54)	3.48 (0.5–9.14)	0.17 (0.03–0.43)	1.38 (0.21–3.47)	2.61 (0.38–7.14)	43.18 (6.37–118.74)	1.95 (0.3–4.96)	17.31 (2.8–45.65)	-3.64(-3.85–3.43)	-3.59(-3.81–3.36)
Western Sub-Saharan Africa	0.01 (0–0.02)	1.49 (0.25–4.22)	0.02 (0–0.05)	1.44 (0.25–4.01)	0.17 (0.02–0.54)	23.63 (3.71–70.29)	0.34 (0.05–1.07)	22.36 (3.59–65.07)	-0.22(-0.31–0.13)	-0.29(-0.37–0.22)

*DALYs* Disability-adjusted life-years, *UI* Uncertainty interval, *SDI* Socio-Demographic Index, *EAPC* Estimated annual percentage change, *ASR* Age-standardized rate, no.: number

Globally, the ASMRs of stroke attributable to LPA experienced a decline from 3.09 per 100,000 persons (95% UI: 0.55–8.09) in 1990 to 2.08 per 100,000 persons (95% UI: 0.41–5.33) in 2019, with an ASMR-related EAPC of -1.61 (95% CI: -1.71–-1.5); the ASDRs of stroke attributable to LPA also experienced a decline from 43.55 per 100,000 persons (95% UI: 7.56–117.17) in 1990 to 31.16 per 100,000 persons (95% UI: 5.69–82.02) in 2019, with an ASDR-related EAPC of -1.35 (95% CI: -1.43–-1.27). Among 21 regions in 1990–2019, the EAPCs in most regions were < 0, while the five highest ASMR- and ASDR-related EAPCs were shared by Southern Sub-Saharan Africa (1.3, 95% CI: 0.68–1.92), Eastern Sub-Saharan Africa (0.58, 95% CI: 0.53–0.62), Southeast Asia (0.56, 95% CI: 0.43–0.69), Central Sub-Saharan Africa (0.23, 95% CI: 0.18–0.27), and Central Asia (0.11, 95% CI: -0.18–-0.4) for ASMR and Southern Sub-Saharan Africa (0.96, 95% CI: 0.36–1.57), Southeast Asia (0.49, 95% CI: 0.4–0.58), Eastern Sub-Saharan Africa (0.34, 95% CI: 0.32–0.37), North Africa and Middle East (0.19, 95% CI: 0.1–0.27), and Oceania (0.05, 95% CI: -0.01–-0.11)for ASDR, respectively (Table 1).

The numbers of deaths and DALYs started to increase in those aged ≥ 50 but decreased in those aged ≥ 85. The majority of deaths and DALYs in 2019 were shared by those aged ≥ 70, with the largest numbers of deaths and DALYs for those aged 80–84. Females shared more deaths and higher levels of DALYs than males among those aged ≥ 75; additionally, both death rates and DALY rates showed an increasing trend in all ages (Fig. 1C and D).

### The national burden of stroke attributable to LPA

**Fig. 2:**
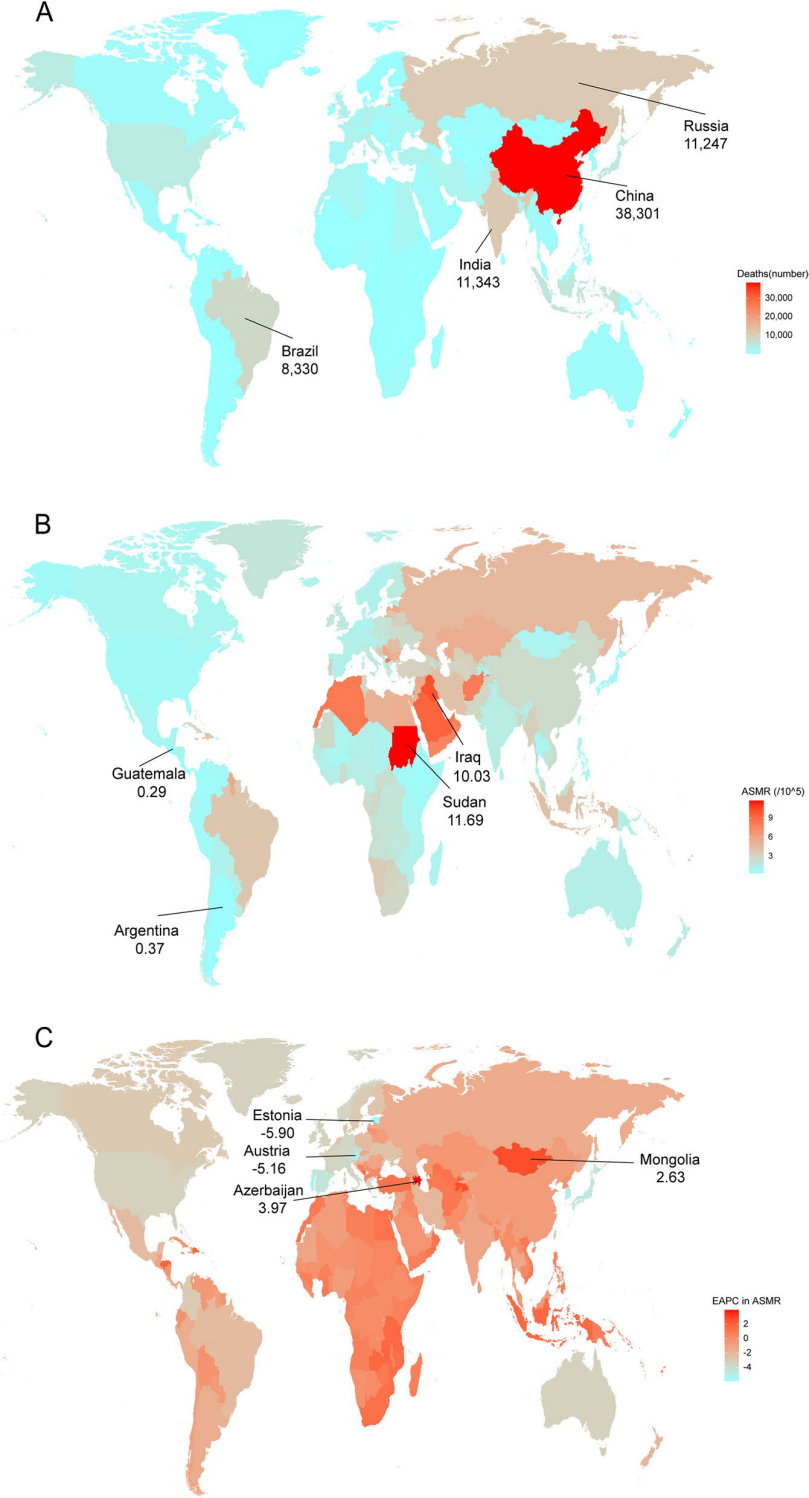
The deaths (**A**), ASMR (**B**), and the ASMR-related EAPC (**C**) of stroke attributable to LPA among 204 countries and territories in 2019. ASMR: age-standardized mortality rate; EAPC: estimated annual percentage change; LPA: low physical activity

**Fig. 3:**
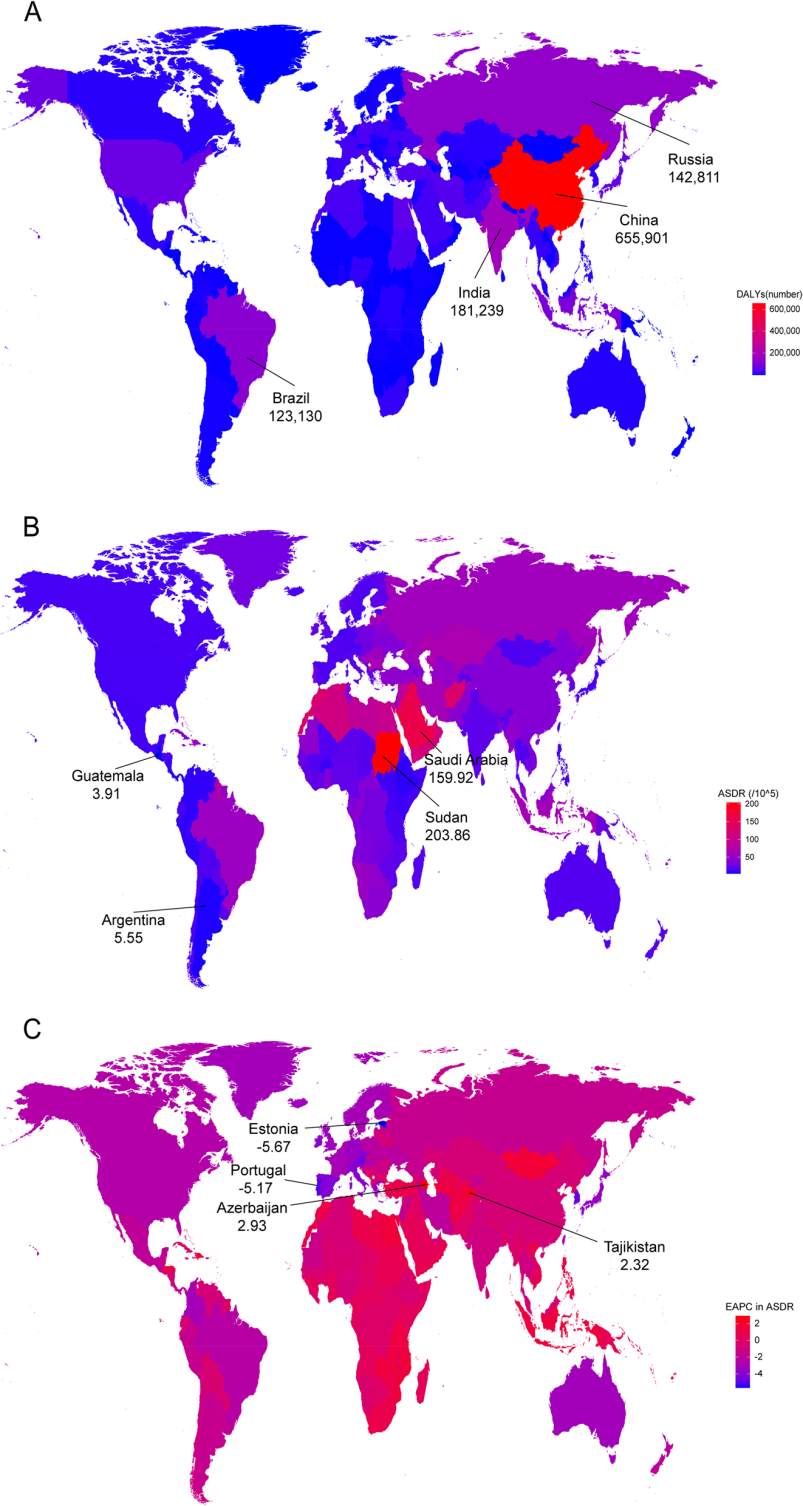
The DALY (**A**), ASDR (**B**), and the ASDR-related EAPC (**C**) of stroke attributable to LPA among 204 countries and territories in 2019. ASDR: age-standardized DALY rate; EAPC: estimated annual percentage change; LPA: low physical activity

Among 204 countries and territories in 2019, the five most deaths were in sequence shared by China, India, the Russian Federation, Brazil, and Indonesia; the five fewest deaths were in sequence shared by Tokelau, Niue, Nauru, Tuvalu, and Palau (Fig. 2A). Similarly, China, India, the Russian Federation, Brazil, and Indonesia shared in sequence the five leading levels of DALY in 2019 (Fig. 3A). Sudan shared the highest ASMR level (11.69 per 100,000 persons (95% UI: 3.36–21.25)) (Fig. 2B) and the highest ASDR level (203.86 per 100,000 persons (95% UI: 52.24–391.56)) (Fig. 3B). A total of 75 countries showed upward trends for ASMRs among most countries with EAPCs < 0 in 1990–2019, wherein Azerbaijan shared the fastest growth (Fig. 2C); similarly, a total of 68 countries showed upward trends for ASDRs, wherein Azerbaijan, Tajikistan, Lesotho, Dominican Republic, and Montenegro shared in sequence the five leading levels of the ASDR-related EAPCs (Fig. 3C).

### The regional and SDI burdens of stroke attributable to LPA

**Fig. 4:**
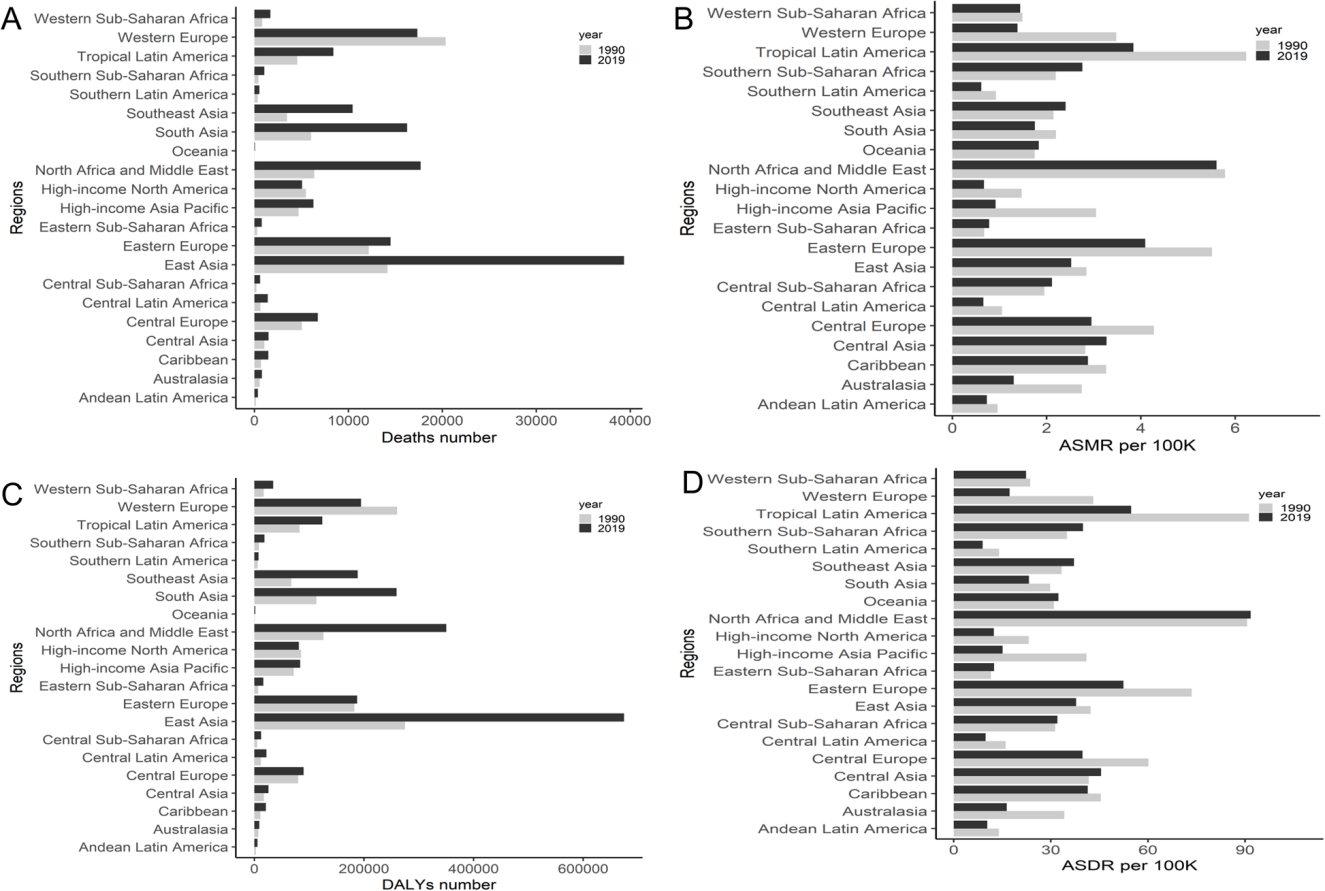
The deaths (**A**), ASMR (**B**), DALY (**C**), and ASDR (**D**) of stroke attributable to LPA in 21 regions. ASMR: age-standardized mortality rate; ASDR: age-standardized DALY rate; DALYs: disability-adjusted life-years; LPA: low physical activity

*East Asia had the most number* of deaths in 2019, with 0.04million (95% UI: 0.006–0.11) or with increases of 177.85% for deaths and 145.38% for DALYs in 1990–2019; Western Europe had the most deaths (0.02million, 95% UI: 0.003–0.05) in 1990. North Africa and Middle East had the highest ASMR in 2019 (5.60 per 100,000 persons (95% UI: 1.34–11.58)) and the second highest ASMR in 1990 (5.79 per 100,000 persons (95% UI: 1.32–12.19)), respectively; Tropical Latin America had the highest ASMR in 1990 (7.43 per 100,000 persons (95% UI: 1.30–15.42)). Among 21 regions, North Africa and Middle East had the highest ASDR in 2019 (91.71 per 100,000 persons (95% UI: 18.75–203.41)) and the second highest ASDR in 1990 (90.62 per 100,000 persons (95% UI: 19.1–204.65)), respectively; Tropical Latin America had the highest ASDR (108.75 per 100,000 persons (95% UI: 17.24–243.98)) in 1990 (Fig. 4).

**Fig. 5:**
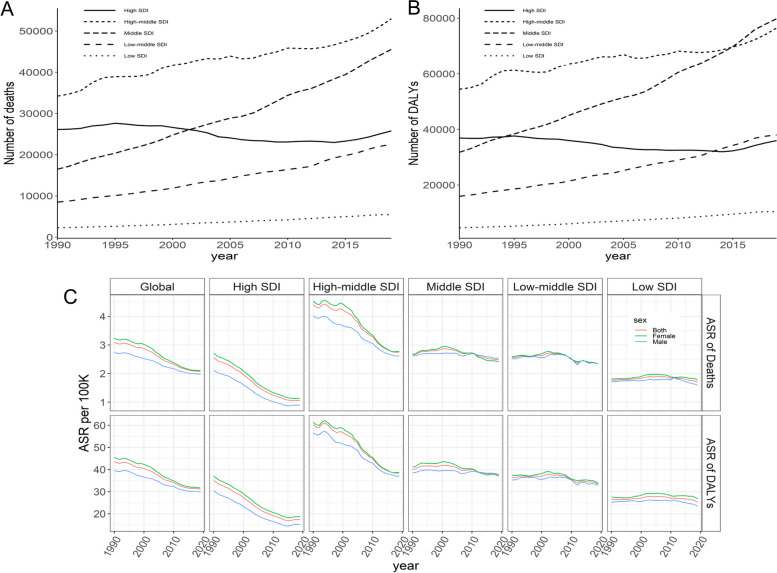
Tendencies in deaths (**A**), DALY (**B**), and their corresponding ASRs (**C**) of stroke attributable to LPA, 1990–2019. SDI: Socio-Demographic Index; ASR: age-standardized rate; DALYs: disability-adjusted life-years; LPA: low physical activity

The number of deaths and DALYs in different SDIs showed great disparities in the past three decades, *with the lowest level of deaths and DALYs* in low SDIs, a flattening off in high and high-middle SDIs, and a stable in high SDIs. Besides those high-middle SDIs with the most deaths in all years, the middle, low-middle, and high SDIs shared an increasing trend in 1990–2019 but a decreasing trend in 2002–2009, respectively. The highest DALYs before 2015 were shared by high-middle SDIs, *although they have been surpassed* by middle SDIs since then. Similarly, the middle, low-middle, and high SDIs shared an increasing trend for DALYs in 1990–2019 but a decreasing trend for DALYs in 1995–2009, respectively (Fig. 5A and B).

For the tendencies of ASMR and ASDR worldwide, ASMR and ASDR showed decreasing trends in 1990–2019; meanwhile, ASMR and ASDR by region fluctuated in the middle, low-middle, and low SDI, wherein females shared higher levels of ASMR and ASDR than males (Fig. 5C).

### Associations of SDIs with ASMR and ASDR

**Fig. 6:**
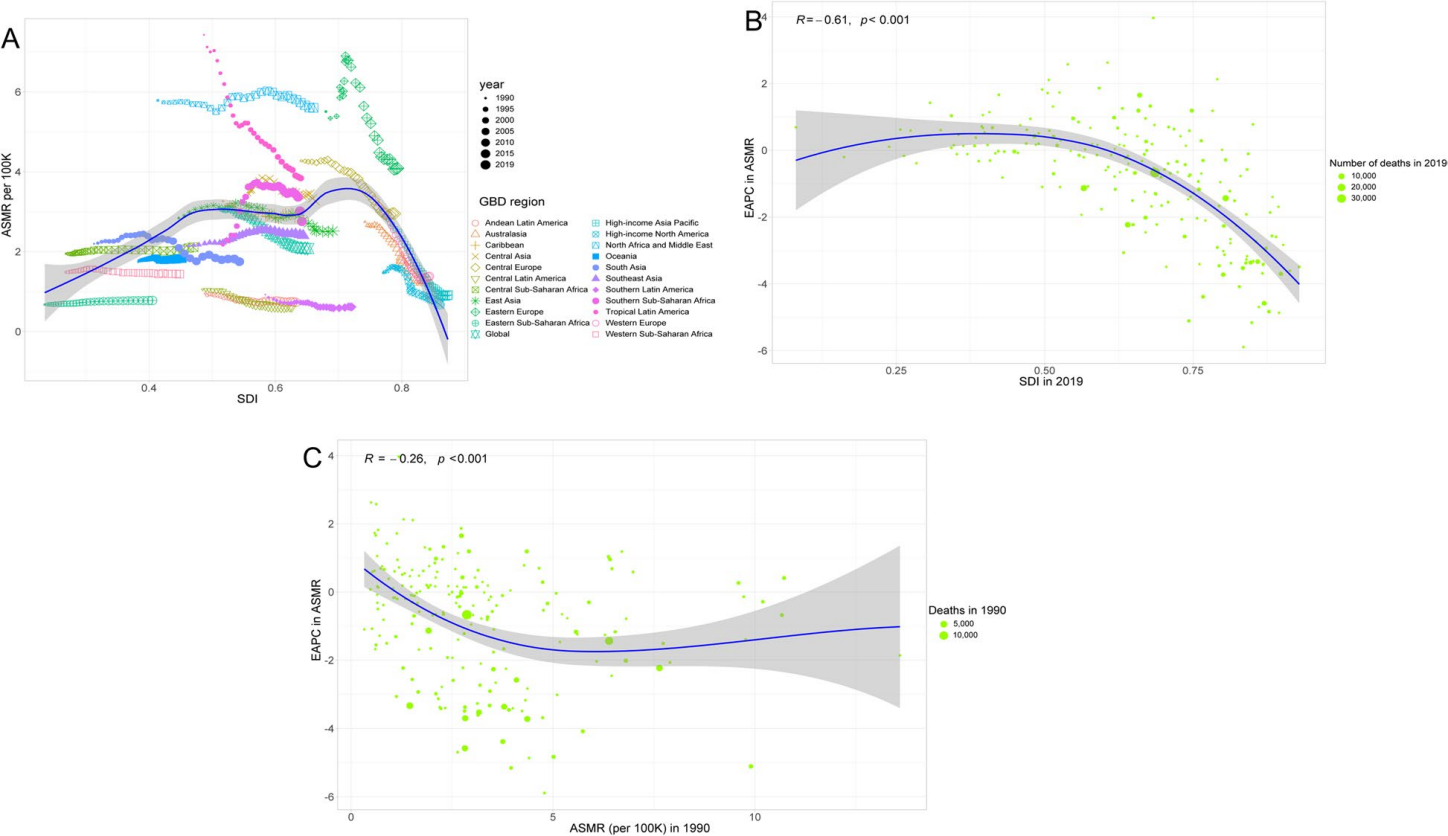
Relationships between ASMR and SDI in 2019 by the GBD region (**A**), the ASMR-related EAPC and SDI across 204 countries in 2019 (**B**), and the ASMR-related EAPC and the ASMR across 204 countries in 1990 (**C**). The points correspond to ASMR in 22 regions, 1990–2019 (**A**) and those across 204 countries and territories (**B** and **C**); a Pearson correlation coefficient and a *P* value were denoted. ASMR: age-standardized mortality rate; SDI: Socio-demographic Index; EAPC: estimated annual percentage change

**Fig. 7:**
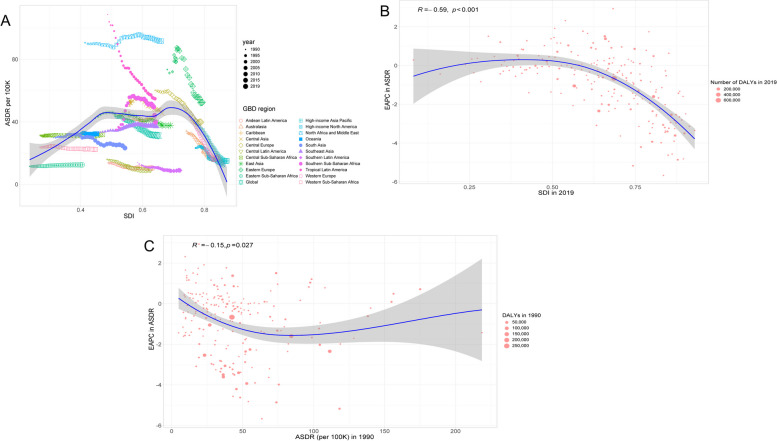
Relationships between ASDR and the SDI in 2019 by GBD region (**A**), the ASDR-related EAPCs and SDI in 2019 (**B**), and the ASDR-related EAPC and the ASDR across 204 countries in 1990 (**C**). The points correspond to ASDR of 22 regions, 1990–2019 (**A**) and those across 204 countries and territories (**B** and **C**); a Pearson correlation coefficient and a *P* value were denoted. ASDR: age-standardized DALY rate; SDI: Socio-demographic Index; EAPC: estimated annual percentage change

SDIs showed an M-shaped association with ASMR for stroke attributable to LPA across different countries, wherein ASMR started firstly to increase but kept a decreasing trend in higher SDIs (Fig. 6A); the ASMR-related EAPC of stroke attributable to LPA was negatively associated with SDI in 2019 (especially within the SDI values greater than 0.5) (R = -0.61, *P* < 0.001) and ASMR in 1990 (R = -0.26, *P* < 0.001), respectively (Fig. 6B and C); such patterns were similar to what ASDR and the ASDR-related EAPC had (Fig. 7).

### Associations of HDIs with ASMR and ASDR

**Fig. 8:**
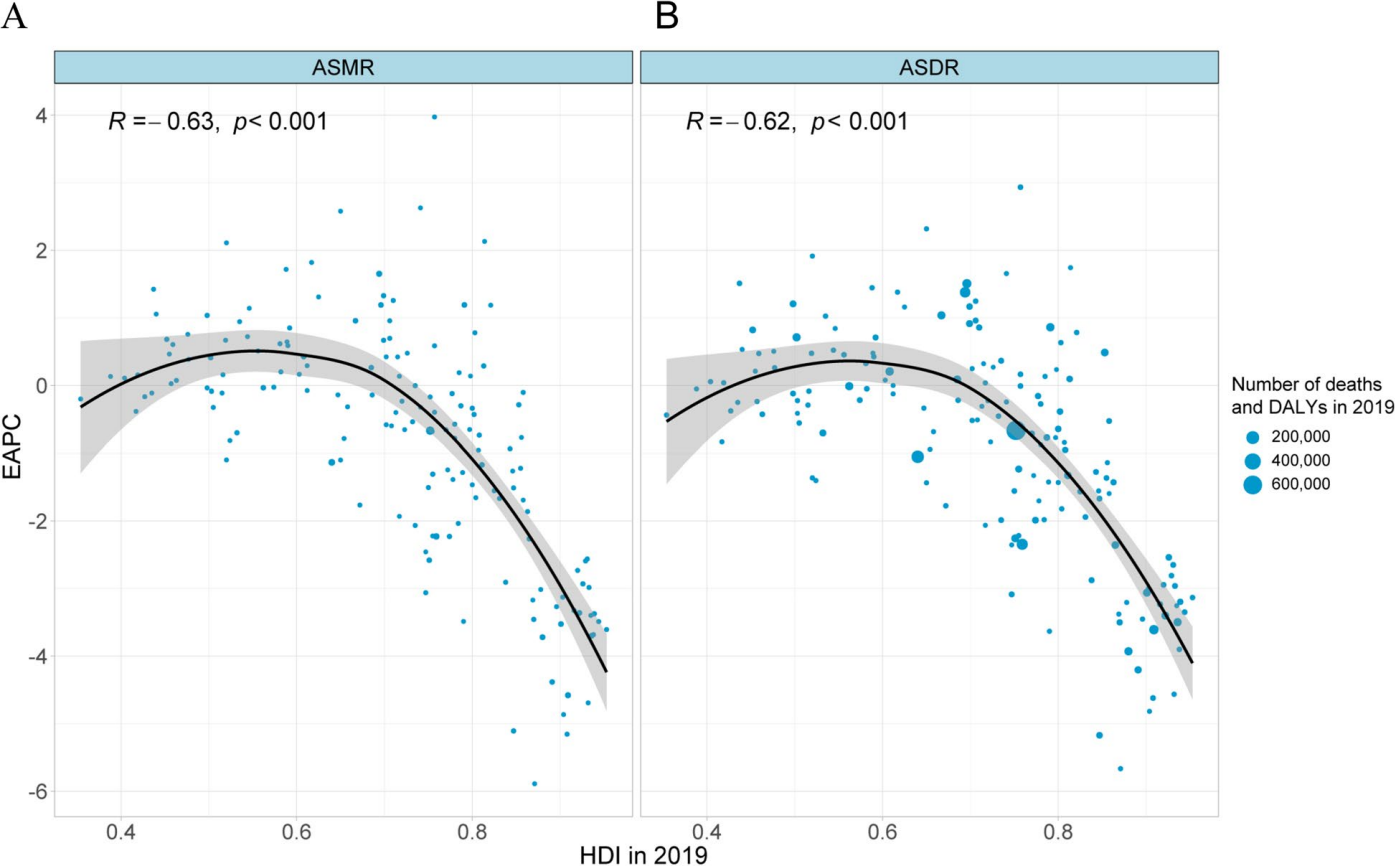
Associations of HDI in 2019 with the ASMR-related EAPC (**A**) and the ASDR-related EAPC (**B**) at national with the Pearson correlation analysis. The points correspond to 158 countries and territories, and the size of the circle increased with the numbers in 2019. ASMR: age-standardized mortality rate; ASDR: age-standardized DALY rate; EAPC: estimated annual percentage change; HDI: human development index

Figure 8 shows associations of HDIs with the ASMR- and ASDR-related EAPCs, including a decreasing trend and a significant association of the ASMR-related EAPC with HDI across different countries in 2019 (R = -0.63, *P* < 0.001) and a significant negative association of the ASDR-related EAPC with HDI across different countries in 2019 (R = -0.62, *P* < 0.001), respectively.

## Discussion

*In this study, we estimated spatiotemporal trends in deaths and DALYs of stroke attributable to LPA at the global, regional, and national levels. We found increases of 1.73-fold for deaths and 1.67-fold for DALYs* but a decline of ASMR and ASDR for stroke attributable to LPA worldwide; additionally, the global burdens of stroke attributable to LPA varied considerably, with the higher rates in North Africa and the Middle East, Tropical Latin America, and Eastern Europe. Our results also showed an M-shaped association of SDI with the burden of stroke attributable to LPA in 2019 (especially with a decreasing trend in high SDIs), a decline and a significant association of the ASMR-related EAPC with HDIs across different countries in 2019, and a significant negative association of the ASDR-related EAPC with HDIs across different countries in 2019, respectively; the ASMR and ASDR in 1990–2019 showed decline in most countries and territories such as Estonia, Portugal, and Austria, besides the increases in the others like Azerbaijan, Tajikistan, and Lesotho.

Physical activity was associated with a decreased risk of stroke in previous studies. Moderate to vigorous exercise *experienced a decline in the risk of st*roke among colorectal cancer survivors in Korea [23]. Similarly, vigorous physical activity was associated with a lower risk of stroke in middle-aged and older adults (especially in males) in China [24]; moderate activity may be the best prevention for stroke in Japan [25]. Different types, frequencies, and intensities of physical activity were associated with a decreased incidence of stroke in the National Health and Nutrition Examination Survey (NHANES) study [26]; longer sedentary times besides low-intensity and moderate-vigorous physical activity were independently associated with an increased risk of stroke [27]; low physical activity was also associated with an increased risk of stroke incidence [28], which is the same as physical inactivity on ischaemic stroke [29]. The pathophysiological mechanism of how physical activity works should exactly be that physical activity is able to strengthen heart function, improve blood vessel function by lowering blood viscosity, platelet aggregation, and thrombosis [30, 31], and promote blood circulation and lipid metabolism by pumping blood into the brain and releasing high-density lipoprotein cholesterol in the blood, respectively [32].

Unlike males being more susceptible to stroke [33, 34], *we observed the different global burdens by gender*, with more deaths and higher levels of DALY, ASMR, and ASDR in females. A potential reason might be that females do fewer physical activities [35, 36]. Nevertheless, males need to be paid more attention based on our findings of deaths and DALYs by gender.

The global burden of stroke attributable to LPA was more severe *in the elderly than the young,* wherein those aged ≥ 70 shared the majority of deaths and DALYs, which mostly support those elderly with high levels of LPA. Accompanying life expectancy increasing continuously [37], the global trend in aging and the burdens of stroke attributable to LPA are growing. Therefore, more physical activities are important for older populations; higher education supply and more policy support are also required.

Burdens of stroke attributable to LPA from 1990 to 2019 varied across the world, with increasing trends in deaths and DALYs in high-middle, middle, low-middle, and low SDI, *wherein the high ASMR and DALY attributable to LPA especially in the countries from the Middle Eastern region and also in the tropical regions (*Figs. 2B and 3B*), and it could be linked to the more sedentary lifestyle or high-temperature environment which is less conducive to outdoor physical activities* [19]; *other* possible reasons are *fewer* demands of physical activities but more changes in recreational and cultural values accompanying with the economic development among those in lower SDI [38]; additionally, *a potential explanation* for those high SDIs with decreasing trends might be high-income countries focusing earlier on promoting physical activity [39]. A stable but decreasing trend in both ASMR and ASDR was shared by the middle, low-middle, and low SDIs and high and high-middle SDIs, respectively; which suggests a picture of improvement in age-standardized impact but rising absolute numbers due to the growing and aging population.

This study included some limitations. First, *information on stroke subtypes was incomplete*, and data quality was not easy to guarantee in low- and middle-income countries. Second, a series of indicators were screened and their corresponding trends in burdens of stroke attributable to LPA worldwide were assessed, other risk factor*s, such as population growth and aging,* were incompletely taken into account. Third, due to analytic data from self-reported questionnaires and *lack of sufficient validity evidence of the participants’ recall and social desirability, some uncertainties of under or overestimated self-report* limited the estimates of physical activity and changes in weekly MET-minutes over time, *accelerometer-measured tool or updated questionnaire should be implemented into these physical activity-related studies in the future.*

## Conclusions

We found increases in deaths and DALYs of stroke attributable to LPA worldwide from 1990 to 2019, with different deaths, DALYs, ASDRs, and ASMRs by gender and region. Thus, *more attention should be paid to* the effects of physical activity on health intervention, and patients with stroke attributable to LPA, especially those aged ≥ 70 and females, should energetically be cared about in the regions of East Asia, North Africa, and the Middle East. Our findings should also help with the development and monitoring of the effectiveness of stroke prevention and management and rehabilitation strategies in different countries and among different populations.

## Data Availability

Data used in this analysis are available from the Global Burden of Disease (GBD) Data Exchange database (http://ghdx.healthdata.org/gbd-results-tool).
